# Effectiveness of the female condom in preventing HIV and sexually transmitted infections: a systematic review and meta-analysis

**DOI:** 10.1186/s12889-020-8384-7

**Published:** 2020-03-12

**Authors:** Alison B. Wiyeh, Ruth K. B. Mome, Phetole W. Mahasha, Eugene J. Kongnyuy, Charles S. Wiysonge

**Affiliations:** 1 Cochrane South Africa, South African Medical Research Council, Cape Town, South Africa; 2 Department of Epidemiology, University of Washington School of Public Health, Seattle, Washington USA; 3 Ottumwa Regional Health Center, 1001 Pennsylvania Avenue, Ottumwa, IA 52501 USA; 4 Grants, Innovation and Product Development, South African Medical Research Council, Cape Town, South Africa; 5 School of Global Health and Bioethics, Euclid University, Banjul, Gambia; 6 Division of Epidemiology and Biostatistics, Department of Global Health, Stellenbosch University, Cape Town, South Africa; 7 Division of Epidemiology and Biostatistics, School of Public Health and Family Medicine, University of Cape Town, Cape Town, South Africa

**Keywords:** Female condom, HIV, Sexually transmitted infections, Systematic review

## Abstract

**Background:**

The effectiveness of female condoms for preventing HIV and sexually transmitted infections (STIs) remains inconclusive. We examined the effects of female condoms on the acquisition of HIV and STIs.

**Methods:**

We searched four databases, two trial registries, and reference lists of relevant publications in October 2018 and updated our search in February 2020. We screened search output, evaluated study eligibility, and extracted data in duplicate; resolving differences through discussion. We calculated the effective sample size of cluster randomised trials using an intra-cluster correlation coefficient of 0·03. Data from similar studies were combined in a meta-analysis. We performed a non-inferiority analysis of new condoms relative to marketed ones using a non-inferiority margin of 3%. We assessed the certainty of evidence using GRADE.

**Results:**

We included fifteen studies of 6921 women. We found that polyurethane female condoms (FC1) plus male condoms may be as effective as male condoms only in reducing HIV acquisition (1 trial, *n* = 149 women, RR 0.07, 95%CI 0.00–1.38; low-certainty evidence). However, the use of FC1 plus male condoms is superior to male condoms alone in reducing the acquisition of gonorrhoea (2 trials, *n* = 790, RR 0.59, 95%CI 0.41–0.86; high-certainty evidence) and chlamydia (2 trials, *n* = 790, RR 0.67, 95%CI 0.47–0.94; high-certainty evidence). Adverse events and failure rates of FC1 were very low and decreased during follow up. Although the functionality of newer female condoms (Woman’s, Cupid, Pheonurse, Velvet, and Reddy) may be non-inferior to FC2, there were no available studies assessing their efficacy in preventing HIV and STIs.

**Conclusion:**

The use of female plus male condoms is more effective than use of male condoms only in preventing STIs and may be as effective as the male condom only in preventing HIV. There is a need for well conducted studies assessing the effects of newer female condoms on HIV and STIs.

**PROSPERO registration number:**

CRD42018090710

## Background

The disease burden resulting from unsafe sex, including human immunodeficiency virus (HIV) infection and other sexually transmitted infections (STIs), has profoundly impacted the health of people in all parts of the world. Globally, it is estimated that 77·3 million people have been infected with HIV since the start of the HIV epidemic, with approximately half of those infected dying from AIDS related illnesses [1]. Although the incidence of HIV has shown a decrease over the last seventeen years, there were 1·8 million newly infected people in 2017 [1]. In sub-Saharan Africa, young women continue to lead in rates of new HIV infection with three in four new infections being amongst young girls aged 15–19 years [1, 2]. A vast majority of new HIV and STI infections in Sub-Saharan Africa occur through heterosexual transmission [3, 4]. In many cases, STIs that go undiagnosed do not only lead to long-term complications such as infertility and cervical cancer but also enhance HIV susceptibility [5, 6]. Consistent condom use remains the most effective barrier against the sexual transmission of HIV. Male condoms have proven to be 80 to 90% effective [7, 8]. Unfortunately, the subordinate status of women in many countries makes negotiating male condom use with partners especially difficult [9, 10]. This makes women particularly vulnerable to HIV infection and other STIs like gonorrhoea, chlamydia, and syphilis. This warrants the need for alternative methods and effective female initiated methods for STI and HIV prevention.

The female condom is a female-initiated dual method of contraception. In 1993, the polyurethane condom also known as FC1 (Female Health Co., Chicago, IL, USA) became the first female condom to be approved by the United States Food and Drug Administration (USFDA) as a contraceptive and a method of protection against STIs, including HIV/AIDS [11]. It was replaced in 2009 by an identical second generation female condom FC2, which is made from synthetic latex and offers the advantage of having a reduced cost of production [12, 13]. Currently, there are four female condoms that have been prequalified by the World Health Organization (WHO)/United Nations Population Fund (UNFPA). These include the Cupid, FC2, Velvet and the Woman’s condom [14]. There are several others that are being developed and in process for UN and FDA approval with the aim of reducing cost and increasing acceptability [15, 16].

Despite variations in the designs of the different types of female condoms that are available, they share common components which include: an outer retention mechanism that prevents invagination, a sheath that lines the vagina, an internal retention mechanism that ensures the condom stays within the vagina and an insertion feature that facilitates insertion of the condom [17]. Current prequalification guidelines recommend that contraceptive efficacy studies be conducted for novel female condom designs that are not considered equivalent to an existing marketed female condom with an established efficacy rate [18]. However, for new female condoms whose design and specifications are sufficiently similar to those of a marketed device with a known efficacy rate established from a clinical effectiveness study, the clinical effectiveness of the new female condom can be established on the basis of a clinical study comparing the incidence of failures modes. Also, the manufacturer may use a device that has been evaluated directly against a device whose effectiveness is known and has been shown to be non-inferior in the event where there is no suitable marketed device available [18].

### Why it is important to do this review

Although laboratory studies suggest that the female condom may be as effective as the male condom in preventing HIV and STIs [19], the evidence remains uncertain especially for the new generation female condoms. Several reviews that have examined the effectiveness of female condoms in preventing STIs and HIV found a limited number of randomized controlled trials [20, 21]. Furthermore, these reviews are outdated and do not examine the functionality of the more recent designs of female condoms that are increasingly being manufactured to address the shortcomings of the older ones. Our review is timely considering the recent reclassification of the female condom from a class III device to a class II device in September 2018 by the USFDA [22]. This reclassification will simplify the regulatory process for the approval of newer female condoms, ensuring that women have more contraceptive options from which to choose. This move has the potential of increasing availability, access, acceptability and uptake of the female condom.

In this systematic review our primary aim was to examine the evidence around the effectiveness of the female condom on the prevention of HIV and other STIs among women. We also assess the functionality of new female condoms compared to the current widely marketed FC2 female condom.

## Methods

### Criteria for considering studies for this review

The methods used to conduct this systematic review are described in the published protocol [23]. We included randomised controlled trials (both individually and cluster randomised) in women engaged in heterosexual activity in any setting, with no clinical or laboratory-confirmed signs of STIs. Included trials compared the female condom with placebo, or other barrier methods for HIV and/or STI prevention. Our outcomes of interest were the Incidence of HIV, and the Incidence of STIs (including but not limited to chlamydia**,** gonorrhoea**,** syphilis**,** herpes simplex virus, trichomoniasis**,***Lymphogranuloma venereum****,*** HPV**,** bacterial vaginosis) and adverse events. We had aimed to assess the effectiveness of the newer female condoms in preventing HIV and other STIs. However, we did not find any published or unpublished studies that assessed this outcome. Available studies mostly assessed the functionality of new types of female condoms, hence we synthesized the evidence around the functionality of these condoms by assessing the condom clinical failure rates as defined by Beksinska et al. 2007 [24]. We included trials in which the FC1 or FC2 were compared top each other, or compared to any other marketed female condoms.

### Search methods for identification of studies

Using a comprehensive search strategy, we searched PubMed, Cochrane Central Register of Controlled Trials (CENTRAL) and EMBASE on the 9 October 2018 with no restrictions. Our search strategy was updated in February 2020. We used the terms ‘female condom’, ‘HIV’, and ‘sexually transmitted diseases’. Details of our search terms can be found in the published protocol. We also searched the reference lists of previous reviews [20, 21], as well as articles included in this review for relevant studies we may have missed through the electronic search of peer-reviewed literature. We searched the WHO International Clinical Trials Registry Platform (ICTRP) and ClinicalTrials.gov for ongoing trials. All identified records were deduplicated using Mendeley reference management software.

### Study selection

Two authors (RKB and EJK, RKB and CSW or AW and PM) independently screened the titles and abstracts obtained from the electronic searches, as well as the full text of all potentially eligible studies for relevance using a standardised eligibility form with predefined inclusion criteria. Disagreements between the authors who assessed study eligibility were resolved by discussion and consensus. Following the eligibility assessment, we classified all studies that met our inclusion criteria as included. Studies that met the design, intervention and participant inclusion criteria but with relevant outcomes not yet available were classified as ongoing (if the study was not yet completed) or awaiting assessment (if the study was completed but the data not yet published and we could not get any relevant outcome data from trial investigators). We excluded studies that did not meet our inclusion criteria and stated the reasons for exclusion.

### Data extraction and management

Two authors independently extracted data using a standardised data extraction form and performed risk of bias assessment. Extracted information included the study details, participant details, intervention details and outcome details. We assessed the risk of bias for RCTs using the Cochrane risk of bias tool for randomized controlled trials as described in the Cochrane Handbook for Systematic Reviews of Interventions [25]. For crossover and cluster RCTs, we assessed for risk of bias specific to these study designs as described in the Cochrane Handbook for Systematic Reviews of Interventions [25]. Disagreements between the authors who extracted data and assessed risk of bias were resolved by discussion and consensus. We planned to asses for publication bias using a funnel plot, but this was not done due to the insufficient number of studies reporting the various outcome measures. Data were entered into Review Manager 5.3 (RevMan 5.3) software and checked for accuracy.

Dichotomous data were presented and compared using risk ratios with 95% confidence intervals. We assessed heterogeneity between trial results by visually inspecting the forest plots for overlapping of confidence intervals, followed by the chi-squared test of homogeneity (with significance defined at an alpha level of 10%). We then used the I^2^ test to quantify the degree of heterogeneity. We analysed the data using RevMan 5.3 and Excel statistical software. We conducted meta-analysis when included studies were similar in terms of participants, interventions, and outcomes. We pooled the study results using the Mantel-Haenszel method and the fixed model effects. When there was substantial heterogeneity, we used the random-effects model. When the I^2^ was greater than 50%, we considered it to be substantial heterogeneity and explored the cause of heterogeneity using subgroup analyses. When the studies were not similar enoiugh to be meta-analysed, we narratively synthesised the evidence.

For cluster randomised trials, we reduced each trial to its effective sample size by dividing its original sample by the design effect; where the design effect equals 1 + (M – 1) * ICC (Higgins 2011). M is the average cluster size and ICC is the intracluster correlation coefficient. We used an ICC of 0·03 which was reported by one of the included studies. Finally, we assessed the quality of the evidence using the Grading of Recommendations, Assessment, Development and Evaluations (GRADE) approach, as outlined in the GRADE handbook [26].

In order to assess non inferiority of new female condoms to widely marketed ones, we used the WHO/UNFPA guidelines for female condom generic specification, prequalification and procurement [18]. As stated in this guideline, included studies were required to have a minimum of 200 women, with at least 5 uses of each condom type. Non inferiority was demonstrated by calculating the difference of total clinical failure rates between the new female condom and the marketed one. The upper bound of the one-sided 95% confidence interval of this difference was expected to be less than 3% for non-inferiority [18]. We conducted both per protocol and intention to treat analysis for all relevant trials using random effects model [27].

## Results

**Fig. 1:**
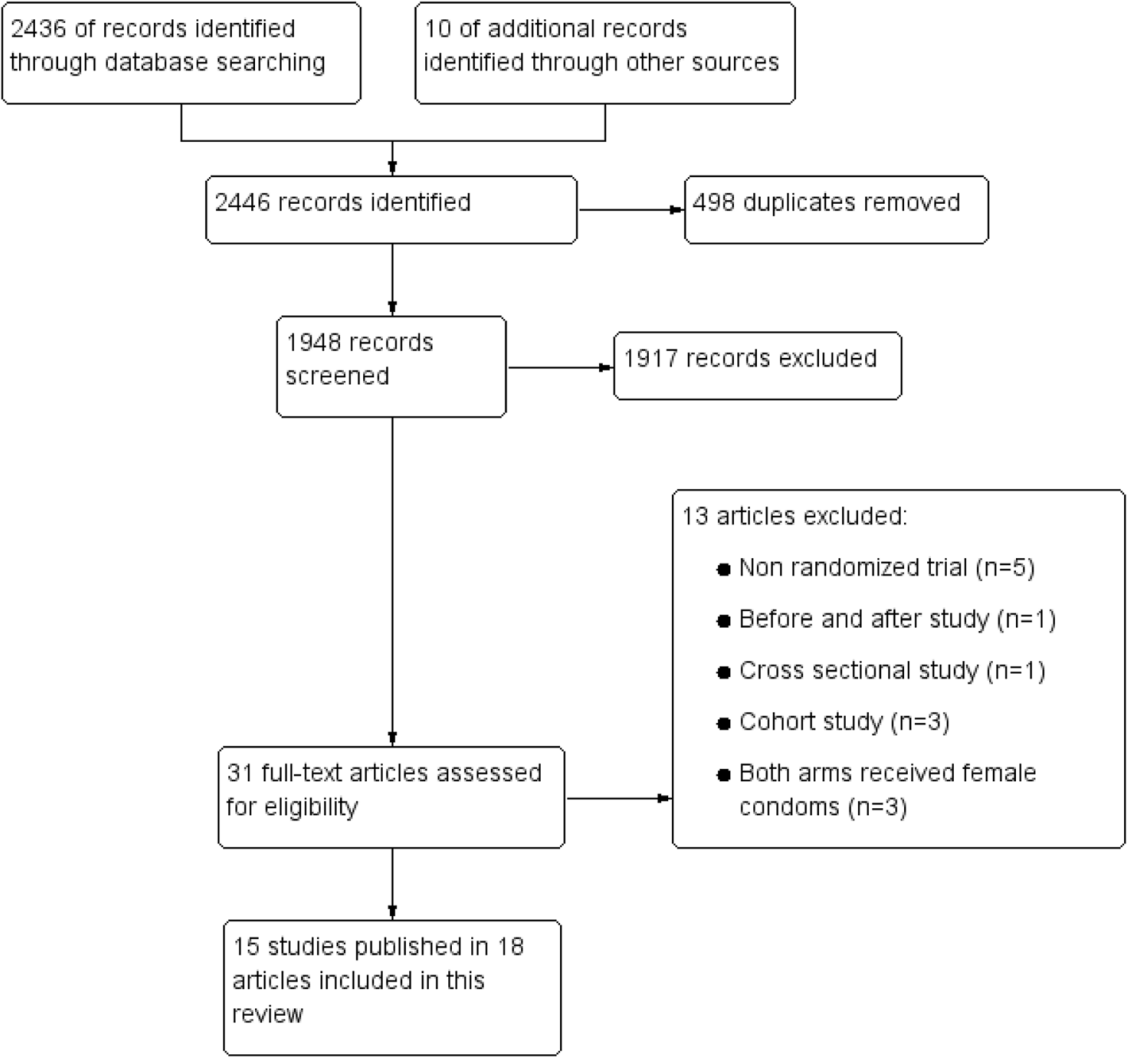
Study flow diagram

Figure 1 summarises our search and selection process. We identified 2325 records during our initial search in 2018. An updated search in 2020 identified 121 new records. We screened 1948 titles and abstracts after removing duplicates. Thirty-one records met our inclusion criteria. Following full text assessment, independent review and discussion of the thirty-one full-text articles, we included fifteen studies published in eighteen articles.

We searched clinicaltrials.gov and the WHO ICTRP and found 4 relevant trial records. Two trials had no comparator arms, the results from one trial had been published and included amongst the included trials, and the results of one completed crossover trial posted in 2010 in which the Program for Appropriate Technology in Health (PATH) Woman’s Condom and the FC2 Female Condom were compared could not be found [28].

### Included studies

#### Study designs

The study designs include: Two cluster randomised trials [29, 30], ten crossover trials including three non-inferiority trials [31–39], and three randomized controlled trials [40, 41]. See Table 1.

**Table 1:** Characteristics of included studies table

Study Id	Study period	Design	Country	Sample size	Participants	Intervention	Comparator	Outcome assessment
FC1 + MC compared to MC								
Fontanet 1998 [29]	1994–1995	Community based cluster randomised trial (sex establishments)	Thailand	548	Women aged over 18 years, female sex workers, not be pregnant, not using a diaphragm, cervical cap, vaginal spermicides or intravenous drugs and should have no evidence of STI	282 women were randomised to FC1 + MC. Participants received training on condom use at baseline	266 women randomised to MC only. Participants received training on condom use at baseline	**Incidence of STI** Chlamydia: Elisa Gonorrhea: culture **Adverse effects** Information from coital logs and questionnaires **Failure modes** Information from coital logs and questionnaires
Feldblum 2001 [30, 42, 43]	Not stated	Community based cluster randomised trial	Kenya	1929	Women aged 18 ± 50 years of age who are not pregnant or desiring pregnancy in the coming year, sexually active and permanent female plantation employees	969 women were randomised to FC1 + MC. Participants received training on condom use at baseline	960 women randomised to MC only. Participants received training on condom use at baseline	**Incidence of STI** Gonorrhoea & chlamydia: ligase chain reaction on urine specimens Vaginal trichomoniasis: InPouchTM TV test system
Ray 2001 [41]	Not stated	Community based randomised trial	Zimbabwe	149	Women aged over 18 who are female sex workers with at least three different paying clients the previous month and residents of Harare	99 women randomised to FC1 + MC	50 women randomised to MC only	**Incidence of HIV** Methods of outcome measurement not described **Incidence of STIs** gonorrhoea: culture Trichomonas: culture Chlamydia: enzyme immunoassay Syphyliis: RPR and TPHA **Failure modes** Information on condom breakage, and reports of invagination and misdirection during interviews **Adverse events** Information on problems, irritation and discomfort during structured interviews
French 2003 [40]	1995–1996	Clinic based randomised trial	USA	1442	Women clinically evaluated on at least one occasion at the main public STD clinic in Philadelphia during the study period. Age range not specified	855 women randomised to FC1 and had access to MCs. They received counselling sessions on the FC	587 women randomised to MC only. They received enhanced MC counselling sessions	**Incidence of STIs** Gonorrhea: culture Chlamydia: Gen-Probe Pace 2C Early syphilis: RPR/MHA or FTA analysis Trichomoniasis: Wet mount microscopy
FC1 compared to MC								
Galvao 2005 [31, 44]	Not stated	Clinic based crossover randomised trial	Brazil	400	Women aged between 15 and 49 years old, sexually active, had not been using condoms as primary birth control method for 1 year or longer and willing to try both FCs and MCs. They had to be able to read the instruction sheet of the FC and MC packages and willing to comply with the study protocol	At different sequences, women were randomly assigned to use FC1 or MCs. Patients were randomly assigned to receive in-clinic educational instructions on condom use or the recommendation to read the condom package inserts.		**Failure modes** Information collected using condom data forms and questionnaires and interviews **Adverse events** Information collected using condom data forms and questionnaires and interviews **Semen exposure** Prostate specific antigen
Macaluso 2007 [32, 44]	2000–2001	Clinic based crossover randomised trial	USA	108	Women aged over 19 years, in a mutually monogamous relationship, with no STIs during the past 6 months, who reported four or more acts of sexual intercourse in the past 30 days	At different sequences, women were randomly assigned to use FC1 or MCs, all within 4 months. They were given a motivational intervention and instructions on condom use and trained on vaginal fluid sample collection		**Failure modes** Information collected using participant filled questionnaires and post-trial interviews **Semen exposure** Prostate specific antigen
FC1 compared to FC2								
Beksinska 2006 [33]	2004	Clinic based crossover randomised trial	South Africa	276	Women at least 18 years of age who are sexually active, not pregnant or nursing, using a hormonal contraceptive method, IUD or sterilized and in good general and genital health. Participants included urban women from family planning, STI and student clinics, rural women attending family planning clinics and commercial sex workers from a sex worker lodge	At different sequences, women were randomly assigned to use FC2 or FC1 with each condom type to be used over a 2–3 months period		**Condom failure modes** Information collected using coital logs and interviews **Adverse events** Information collected using coital logs and interviews
FC1 compared to new female condoms								
Schwartz 2008 [39]	2004–2005	Clinic based Crossover randomised trial	USA	75	Couples ≥18 yrs. with low risk for HIV or STIs without any known sensitivities or allergies to study products or prior experience with FCs. Those at risk for pregnancy used a steroidal contraceptive	At different sequences, women were randomly assigned to use Woman’s condom or FC1 with each condom type to be used over a 2–4-week period. 3-mL package of lubricant and instructions for use was distributed alongside the Woman’s condom		**Condom failure modes** Self-reported information collected using dairies and questionnaires **Adverse events** Self-reported information collected using dairies and questionnaires
FC2 compared to new female condoms								
Hou 2010 [37]	2007	Community based crossover randomised trial	China	291	Women who are female sex workers. Women with an STI, allergies or known sensitivities to polyurethane silicone lubricants, or vaginal lubricants and prior experience with FCs were excluded	At different sequences, women were randomly assigned to use Pheonurse condoms or FC2. An insertion tool, a water-based lubricant, sanitary towels, and disposal bags were provided in the Phoenurse packaging		**Condom failure modes** Information collected using daily recording forms given to participants as well as questionnaires
Joanis 2011 [38]	2007–2008	Clinic based crossover randomised trial	South Africa	170	Women aged over 18 years, with no known allergies to the study products, using a reliable, non-barrier method of contraception, free of STIs, sexually active and monogamous. They should not be practicing sex workers and should not be pregnant or breastfeeding	At different sequences, women were randomly assigned to use Woman’s condom, V-amour condoms or FC2 with each condom type to be used over a 3 week period. During the Woman’s condom use period, participants were also provided with five packages of water-based lubricant		**Condom failure modes** Information collected using coital logs and interviews **Adverse events** Information collected using coital logs and interviews
Beksinska 2013 [34]	2011–2012	Clinic based non-inferiority crossover randomised trial	China and South Africa	600	Women aged 18 to 45 years, with no known allergies to the study products, using a reliable, non-barrier method of contraception, free of STIs, sexually active and monogamous. They should not be practicing sex workers and should not be pregnant	At different sequences, women were randomly assigned to use Woman’s condom, Condom Feminine, Cupid or FC2. Woman’s condom was supplied with a water-based lubricant		**Condom failure modes** Information collected using coital logs and interviews **Adverse events** Information collected using coital logs and interviews
Beksinska 2015 [35]	2013–2014	Clinic based non- inferiority crossover randomised trial	South Africa	300	Women aged 18–45 years, no known allergies to study products, using a reliable non barrier method of contraception, free of STIs, sexually active and monogamous. They should not be practicing sex workers and should not be pregnant	At different sequences, women were randomly assigned to use Velvet, Cupid 2 or FC2		**Condom failure modes** Information collected using coital logs and interviews **Adverse events** Information collected using coital logs and interviews
Beksinska^a^ 2018 [36]	2017	Crossover randomised trial	South Africa	55	Women with mean age of 28 years, with ≥10 years of schooling	At different sequences, women were randomly assigned to use Wondaleaf condom or FC2		**Condom failure modes** Methods of outcome measurement not described
Beksinska 2019 [45]	2015–2016	Clinic based non-inferiority crossover randomised trial	South Africa	278	Sexually active monogamous women, 18 to 45 years, with no known allergies to the study products, using a reliable, nonbarrier method of contraception, and free of STIs. Pregnant women were excluded.	At different sequences, women were randomly assigned to use modified Woman’s condom (WC2) or FC2		**Condom failure modes** Information collected using coital logs and interviews **Adverse events** Information collected using coital logs and interviews
Cheng 2019 [46]^a^	Not stated	Randomised trial	Not stated	300	Not stated	Women were randomly assigned to use either China made FC (FCc) or USA made FC (FC2)		**Condom failure modes** Method of data collection not described

^a^ Conference Poster/Abstract

*FC1* Polyurethane female condom; *FC2* Synthetic latex prototype female condom; *FTA* Fluorescent treponemal antibody absorption; *FC* Female condom

*MHA* Microhemagglutination assay; *MC* Male condom; *TPHA* Treponema Pallidium Hemagglutination; *RPR* Rapid Plasma Reagin

#### Participants

The number of participants in each study ranged from 55 to 1929 women over 15 years old. The fifteen studies included a total of 6921 women. Accounting for the design effect in the two cluster-randomised trials with 2477 participants (using an intra-cluster correlation coefficient of 0·03 reported by one of them) their combined effective sample size becomes 790 participants.

The included studies were mostly conducted in women in low-income and middle-income countries: South Africa (*n* = 5), China (*n* = 1), China and South Africa (*n* = 1), Thailand (*n* = 1), Kenya (*n* = 1), Zimbabwe (*n* = 1) and Brazil (*n* = 1). In one study (an abstract), the authors did not mention the country where the study was conducted [46]. Three trials were conducted in high income setting, all in the USA. The women in these studies included both those considered to have a high risk of HIV/STI transmission such as female sex workers, and women in monogamous relationships judged as having a low risk. See Table 1.

#### Interventions

Four trials compared the polyurethane female condom (FC1) plus male condom to the male condom only. In three trials, participants in the intervention group were given a combination of FC1 and male condoms [29, 30, 41], In the fourth trial, the women in the intervention group were given FC1 and had access to male condoms [40]. Two crossover studies compared the female condom to the male condom. At different sequences, women were randomly assigned to use FC1 or male condoms [31, 32]. The focus of these two studies was mainly to assess mechanical problems and semen exposure while using female condoms.

In eight crossover trials, the functionality of new female condoms was compared against widely marketed ones; one trial compared FC2 to FC1 [33], the Woman’s condom was compared to the FC1 in one trial and in seven trials the FC2 was compared to new female condoms. More information is provided in Table 1.

#### Outcomes

The main outcomes reported in studies that compared the female plus male condom to the male condom only were incidence of STI, mechanical problems and adverse events. Only one study reported on the effect of female plus male condoms on HIV transmission [41]. In two studies, semen exposure was measured using prostate specific antigen [31, 47].

Amongst studies that compared the functionality of new female condoms against widely marketed ones, female condom failure modes were the main outcomes reported. The failure modes reported were breakage, slippage, misdirection, invagination, total clinical failure and total failure rates.

### Excluded studies

We excluded eleven articles for the following reasons: non randomized trial (*n* = 5) [47–51], before and after study (*n* = 1) [52], cross sectional study (*n* = 1) [53], cohort study (*n* = 3) [54–56], both arms received female condoms and it was not the main intervention (*n* = 3) [42–44]. See Fig. 1.

### Risk of bias in included studies

We assessed the included studies for selection bias. One study assigned participants to intervention arm based on the week of initial visit and this was scored as high risk for random sequence generation and unclear risk for allocation concealment [40]. Three studies failed to provide sufficient information and were scored as having unclear risk of bias for both random sequence generation and concealment [31, 36]. Three studies reported adequately on the methods of random sequence generation but failed to report on allocation concealment and were scored as having unclear risk of bias for allocation concealment [29, 30, 37]. The rest of the studies adequately reported random sequence generation and allocation concealment and were judged to have low risk of bias for both domains.

With regards to blinding, it was generally difficult to blind the participants and to some extent the research personnel due to the nature of the female condom. All studies had unclear risk of bias for blinding of participants and personnel. All except one study had unclear risk of bias for blinding of outcome assessors [40].

There was high risk of attrition bias in three studies; Macaluso 2007 reported attrition rates of 42% [32] In the study by French 2003, only 50·2% of the female/male condom arm and 51·1% of the male condom only arm completed at least one visit [40]. Fontanet 1998 reported that forty-four women had no follow-up at all, 11 (4·3%) from the male condom group, and 33 (11·7%) from the male/female condom group. Almost half of the study participants were lost to follow-up after 3 months [29]. Six studies were judged as having low attrition rates [30, 34, 35, 38, 39, 45] and the rest were rated as unclear. Three studies were rated as having low risk of reporting bias, with the rest providing insufficient information to enable judgement, hence judged to have unclear risk of bias.

### Other potential sources of bias

In four studies, the authors reported support from companies that manufacture female condoms including; Chartex international [29, 41], the Female Health Company [40], and Cupid Ltd. [35] The support included free condom supplies in one study and funding in three studies and both condom supplies and funding in one study. Details about how potential conflicts of interest were handled are not described. We rated these studies as having unclear risk of bias due to lack of enough information to make a definitive judgement. Two studies did not provide information regarding funding and were also considered to have unclear risk of bias. We had planned to assess for publication bias using a funnel plot. This was not done due to the small number of studies.

In cross-over trials, participants randomly receive a sequence of different treatments. These trials are prone to carryover bias which occurs when participants are switched from one intervention to another without an adequate washout period in-between. Due to the nature of the intervention in this review, we judged the potential for carryover bias to be low. We have summarised the risk of bias in included studies in Table 2.

**Table 2:**
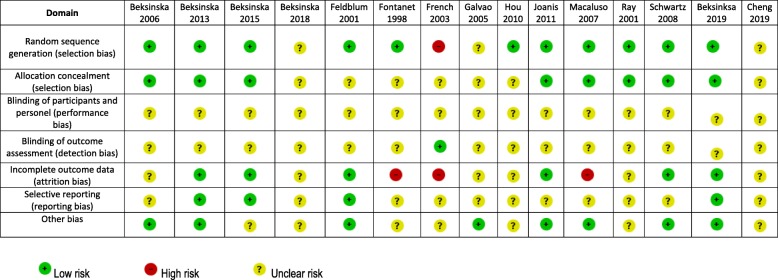
Summary of risk of bias in included studies

Low risk, High risk, Unclear risk.

### Effects of female condoms on HIV and other STIs

#### Comparison of the female (plus male) condom to the male condom only

All studies included in this comparison used the polyurethane female condom also known as the FC1.

### Incidence of HIV

One study including 149 participants compared the effect of the female plus male condom to the male condom only on the incidence of HIV infection [41]. This was a randomized controlled trial conducted in Zimbabwe amongst female sex workers. During the study, no woman in the female plus male condom group became HIV infected, while three women became HIV positive in the male condom only group. However this difference was not statistically significant (RR 0·07, 95% CI 0·00 to 1·38).

### Incidence of STIs

Four studies that compared the female plus male condom to the male condom, reported on this outcome [29, 30, 40, 41]. Three of these studies compared the female plus male condom to the male condom only [29, 30, 41] and one compared the female condom alone to the male condom though the authors report that over one third of the women in the female condom group had access to and used the male condom [40]. Three of these trials, including 2232 women reported this outcome in a manner that was similar enough to be combined.

**Fig. 2:**
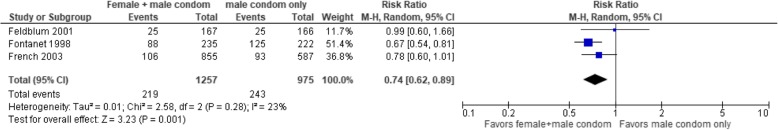
Forest plots of comparison male + female condom versus male condom on the risk of STI

As seen in Fig. 2, the use of the female plus male condom significantly reduces the risk of any STI when compared to male condom only (RR 0·74, 95% CI 0·62 to 0·89; I^2^ = 23%). When the data were disaggregated, the use of female plus male condoms compared to male condoms only significantly reduced the risk of gonorrhoea (2 trials, *n* = 790 participants, RR 0·59, 95%CI 0·41 to 0·86) and chlamydia (2 trials with 790 participants, RR 0·67, 95%CI 0·47 to 0·94). The pooled effect estimate was not significant for trichomoniasis (2 trials with 790 participants, RR 1·01, 95%CI 0·63 to 1·60) and genital ulcer disease (1 trial with 475 participants, RR 4·72 95% CI 0·23 to 97·87).

### Adverse events and condom failure

Three studies reported adverse events. Ray 2001 found that 14% of the participants reported problems, irritation or discomfort with the use of the female condom [41]. Fontanet 1998 reported that the female condom was well tolerated, with no reports of genital lesions that could be attributed to the use of the female condom [29]. Galvao 2005 evaluated the frequency of self-reported acceptability problems. These included non-menstrual vaginal bleeding during intercourse, pain or discomfort felt by either the male or the female partner and noise made by the condom during use. The frequency of acceptability problems was higher in the female condom group [31].

None of the six included studies that compared the male to the female condom reported on the total clinical failure rates or total condom failure rates. However, four studies reported on at least one of the four categories of failure: breakage, invagination, misdirection and slippage. Due to heterogeneity in the reporting of condom failure modes between the included studies, the findings could not be meta-analysed. Two studies reported on breakage; Fontanet 1998 and colleagues reported that male condoms tore more often than female condoms (2·8% compared to 0·5%) [29]. Similarly Macaluso 2007 found that when compared to the male condom, the female condom had lesser reported cases of breakage (0·3% vs 1·3%) [32]. With regards to slippage, both Fontanet 1998 and Macaluso 2007 found that slippage rates during use were higher for the female condom than the male condom: 5·7% versus 1·3% and 10·6% vs 2·1 for complete slippage, 2·6% for partial slippage ≥1 in. and 1·7% for partial slippage ≤1 in. respectively [29, 32]. The authors further highlight that the proportion of female condoms slipping in or out decreased during follow up visits. Two studies found that the rates of invagination for the female condom were generally low. Ray 2001 reported invagination in 3·4% of cases and Macaluso 2007 reported a rate of 3% [32, 41]. Three studies reported on misdirection, which was generally low. Fontanet 1998 and Ray 2001 each reported misdirection in 3.0% of cases amongst those who used the female condom and Macaluso 2007 reported a rate of 5·6% [29, 32, 41]. Galvao 2005 compared the rates of mechanical problems between the male and female condom groups [31]. Male condom mechanical problems were grouped together and included breakage during intercourse, total or partial slippage either during intercourse or during withdrawal and semen leakage on the woman’s body. Female condom mechanical problems were equally grouped together and included; breakage during intercourse, slippage, misdirection, invagination, semen leakage onto the woman’s body, condom clung to penis, moving with it during intercourse and problems encountered by the participants with the inner ring during intercourse. In this study, female condoms had significantly higher rates of self-reported mechanical problems than male condoms (20% vs 12%). For each condom type, women who received in-clinic educational instructions on the female and male condoms reported lesser mechanical problems compared with those who had to read the condom package inserts (FC: 6% vs 14% and MC: 4% vs 8%).

### Functionality studies

#### Comparison of the FC2 to the FC1

One crossover trial conducted in Durban South Africa and including 276 women, compared the functionality of FC2 to FC1 [33]. Each study participant was required to use ten of each condom type with their partners within a 2 to 3 month study period. Approximately one quarter of the study participants were lost to follow up during the study. Functionality measures assessed were breakage (clinical, nonclinical and total), incorrect penetration, outer ring displacement (partial, complete and total), and slippage (partial and complete). Though twice as many women reported incorrect penetration with FC1 than FC2, there was no statistically significant difference in the total clinical failure rates between both condoms (RR 0·81, 95% CI 0·61 to 1·08). The number of participants reporting discomfort and adverse events including; discomfort during and after insertion, pain after insertion before sex, pressure causing urge for micturition, discomfort during sex, device uncomfortable to use and bleeding were similar in both groups. There was a non-significant excess number of cases of burning/rash or itching in the FC2 group (RR 11·00, 95% CI 0·61 to 198·83). The effect on HIV incidence is not reported. One case of STI, presenting as a white discharge and confirmed using syndromic management was reported in the FC1 group (RR 0·33 95% CI 0·01 to 8·18) [33].

Though the authors did not state that this was a non-inferiority trial, it was powered enough to establish non inferiority. The difference of the total clinical failure rates of the FC2 compared to FC1 met the criteria for non-inferiority when we used both per protocol and Intention to treat analysis (RD -0·01, 95% CI − 0·02 to 0·00).

#### Comparison of new female condoms to the FC2 and the FC1

One study including 75 women compared the FC1 to the Woman’s condom [39] and seven studies including 1994 women compared the FC2 condom to other types of female condoms [34–38, 45, 46]. The Woman’s condom resulted, in non-significantly lower rates of total clinical failure (RR 0·72 95% CI 0·51 to 1·02) when compared to the FC1. However, there was a significantly lower rates of total condom failure (RR 0·74 95% CI 0·59 to 0·93) and adverse events (RR 0·28 95% CI 0·17 to 0·46). In addition to the standardised definition of failure modes [24], the author’s definition of clinical failure rates in this trial includes other measures such as the partial turning inside out of the female condom as their measures of female condom performance were adapted from those of the male condom [39, 57]. This study was not powered enough to establish non-inferiority.

**Fig. 3:**
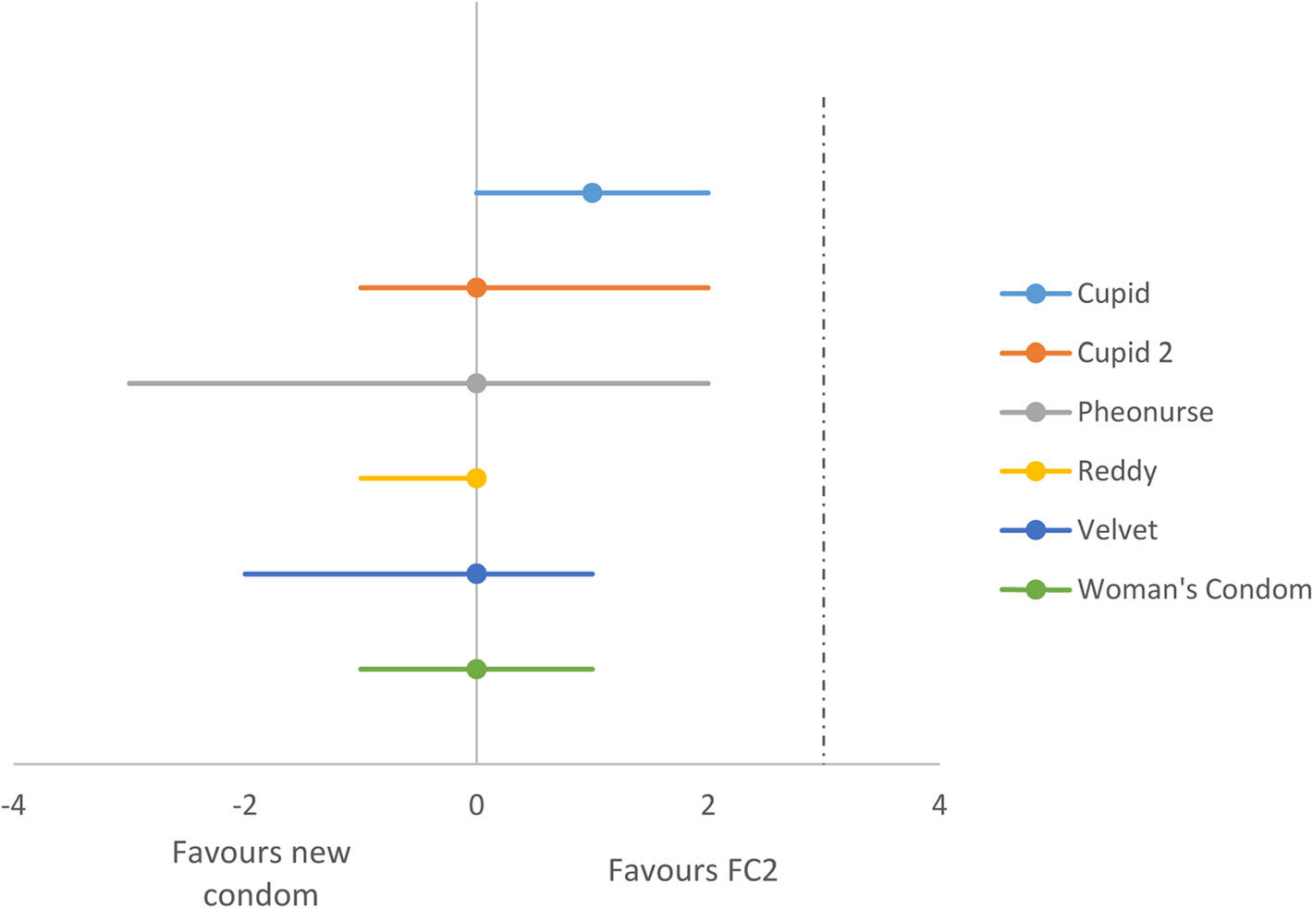
Non-inferiority of new female condoms compared to the FC2 showed using estimates of the risk differences in the total clinical failure rates

When we considered the seven studies that compared the new female condoms to the FC2, there was no significant difference in the total clinical failure rates when the cupid/cupid 2 condoms (2 trials, *n* = 900, RR 1·22 95% CI 0·98 to 1·52) [34, 35], Velvet condom (1 trial, *n* = 300, RR 0·90 95% CI 0·63 to 1·29) [35], Woman’s condom/Woman’s condom 2 (3 trials, *n* = 1058, RR 0.84 95% CI 0·68 to 1·04) [34, 38, 45], Reddy female condom (2 trials, *n* = 770, RR 0·85 95% CI 0·65 to 1·11) [34, 38], and Pheonurse (1 trial, *n* = 291, RR 1·00 95% CI 0·92 to 1·08) [37] were compared to the FC2. One conference abstract for a trial comparing the Wondaleaf condom to the FC2 reported total clinical failure rates of 5·3% and 7·5% respectively [36]. Another abstract describing a study in which the functionality of the China female condom (FCc) was compared to the FC2 reported total clinical failure rates of 0.9 and 1.1% for the FCc and the FC2 respectively [46]. The difference in total clinical failure rates was deemed non-significant. The full text of this article was unavailable. We examine the risk difference and found that the functionality of the cupid, cupid, Velvet condom, Woman’s condom, Reddy and phoenurse may be non-inferior to the FC2 based on the clinical failure rates, without any differences observed in per protocol and intention to treat analysis (see Fig. 3). The trial comparing the Wondaleaf condom to FC2 was not powered enough to demonstrate non-inferiority.

Four studies compared the total condom failure rate between the FC2 and other types of female condoms. There was no significant difference in total female condom failure rates when the Cupid/Cupid2 condoms (2 trials, *n* = 900, RR 1·16 95% CI 0·96 to 1·40) [34, 35], Velvet condom (1 trial, *n* = 300, RR 0·88 95% CI 0·66 to 1·18) [35], Woman’s condom (2 trials, *n* = 878, RR 0.84 95% CI 0·49 to 1·46) [34, 45], Reddy female condom (1 trial, *n* = 600, RR 0·87 95% CI 0·65 to 1·15) [34] were compared to the FC2. None of the studies that compared the new female condoms to the FC2 assessed their direct effects on the incidence of HIV infection or STIs.

## Discussion

### Summary of the main results

In this review, we aimed to assess the effects of the female condom in preventing HIV and other STIs among women. Available evidence suggest that there may be no difference in the incidence of HIV infection when women used the polyurethane female condom plus the male condom compared to those who used the male condom only. However, female plus male condom reduces the risk of gonorrhoea and chlamydia and probably reduces the risk of trichomonas and other STIs when compared to the male condom only. Adverse events associated with use of the female condom were generally few and not life threatening. Condom breakage was more frequently reported amongst users of the male condom and slippage amongst users of the female condom. The rates of invagination and misdirection of the female condom were low ranging from 3 to 3.4% and 3–5.6% respectively.

We note however that there were no eligible studies assessing the effectiveness of the new female condoms in preventing HIV and STIs. Available studies assessed the functionality of these new condoms against existing ones for the purpose of prequalification.

One study comparing the functionality of the FC2 to the FC1 found it to be non-inferior. The cupid, cupid 2, Velvet, Woman’s, Reddy and Pheonurse, condoms were non-inferior to the FC2 based on their total clinical failure rates. Functionality studies which are often required for prequalification showed that FC2 was equivalent to FC1 based on non-clinical and functionality data, hence a full contraceptive efficacy study on FC2 was not necessary. However, this may not be the case for many of the new types of female condoms which, warranting the need for contraceptive efficacy studies.

Data from self-reports show that the usage of the female condom was generally poor in the intervention arms. French et al. in a sub study found that male condoms accounted for one third of the condom protected sex acts in the female condom group [40]. Feldblum et al. also highlight that though female condom distribution increased in the female plus male condom group, 39 and 58% of the women reported never using the female condom at 6 months and 12 month follow up visits [29]. Fontanet 1998 instructed women to use the female condom as a backup when clients refused to use male condoms and the use of the female condom remained limited, accounting for only 12% of all sexual acts [29]. Ray found that though the proportion of women in the female + male condom group who consistently used the male condom increased, only 3–9% of women reported consistently using the female condom with clients during the study [41]. This further highlights the importance of high-quality studies assessing the acceptability of female condoms in the targeted populations.

### Certainty of the evidence

The strength of this review lies in our adherence to the standardised guidelines on the conduct and reporting of systematic reviews [25]. We used the GRADE approach to assess the certainty of the evidence as shown in the summary of findings table 3 [26]. We judged the quality of evidence for FC1 plus male condoms compared to male condoms only as low and moderate in reducing the risk of HIV and STIs respectively. The certainty of the evidence was downgraded for very severe imprecision in the case of HIV and high risk of bias in the case of STIs. We rated the certainty of the evidence for the effect of FC1 plus male condom in preventing gonorrhoea and chlamydia as high. The effects of the FC1 plus male condom in preventing genital ulcer disease and trichomoniasis were rated as moderate. In both instance, we downgraded for severe imprecision.

**Table 3:** Summary of findings table Female condom + male condom compared to Male condom only for prevention of HIV an STIs Patient or population: prevention of HIV an STIs Intervention: Female condom + male condom Comparison: Male condom only

Outcome № of participants (studies)	Relative effect (95% CI)	Anticipated absolute effects (95% CI)			Certainty
				Difference	
HIV infection № of participants: 149 (1 RCT)	RR 0.07 (0.00 to 1.38)	6.0%	0.4% (0 to 8.3)	5.6% fewer (6 fewer to 2.3 more)	⨁⨁◯◯ LOW ^a^
Any STI № of participants: 2232 (3 RCTs)	RR 0.74 (0.62 to 0.89)	24.9%	18.4% (15.5 to 22.2)	6.5% fewer (9.5 fewer to 2.7 fewer)	⨁⨁⨁◯ MODERATE ^b^
Gonorrhoea № of participants: 790 (2 RCTs)	RR 0.59 (0.41 to 0.86)	15.2%	9.0% (6.2 to 13.1)	6.2% fewer (9 fewer to 2.1 fewer)	⨁⨁⨁⨁ HIGH
Chlamydia № of participants: 790 (2 RCTs)	RR 0.67 (0.47 to 0.94)	16.0%	10.7% (7.5 to 15)	5.3% fewer (8.5 fewer to 1 fewer)	⨁⨁⨁⨁ HIGH
Trichomonas № of participants: 790 (2 RCTs)	RR 1.01 (0.63 to 1.60)	8.0%	8.1% (5 to 12.8)	0.1% more (3 fewer to 4.8 more)	⨁⨁⨁◯ MODERATE ^a^
Genital ulcer disease № of participants: 457 (1 RCT)	RR 4.72 (0.23 to 97.87)	0.0%	0.0% (0 to 0)	0.0% fewer (0 fewer to 0 fewer)	⨁⨁⨁◯ MODERATE ^a^

*CI* Confidence interval; RR: Risk ratio

*GRADE* Working Group grades of evidence

High certainty: We are very confident that the true effect lies close to that of the estimate of the effect

Moderate certainty: We are moderately confident in the effect estimate: The true effect is likely to be close to the estimate of the effect, but there is a possibility that it is substantially different

Low certainty: Our confidence in the effect estimate is limited: The true effect may be substantially different from the estimate of the effect

Very low certainty: We have very little confidence in the effect estimate: The true effect is likely to be substantially different from the estimate of effect

Explanations

^a^Includes only one study with small sample size and wide confidence intervals

^b^High risk of selection bias due to lack of proper randomization in one of the trials

The certainty of the evidence from studies comparing the Woman’s condom to the FC1, and the Wondaleaf condom to the FC2 was judged to be of low mostly due to severe imprecision resulting from the small number of study participants. The certainty of the non-inferiority analysis comparing the functionality of the FC2 to the FC1 and the cupid, cupid 2, Velvet, Woman’s, Reddy, Pheonurse, condoms to the FC2 was rated as moderate, downgrading for imprecision.

### Agreements and disagreements with other studies or reviews

Several reviews have attempted to answer our review question [12, 20, 21]. Minnis and colleagues in 2004 reviewed the evidence around the effectiveness of the female condom in preventing STIs from three randomized trial and one prospective cohort study. They reported that results from these studies provide evidence that female condom confer as much protection from STIs as male condom [20]. A review by Gallo et al. in 2013 examined the evidence from randomized and non-randomised studies. They argue that although female condoms (or a combination of female plus male condoms) may provide similar degrees of protection against pregnancy and STIs as do latex male condoms alone, they called for more comparative research to demonstrate this [12]. Vijayakumar and colleagues in 2006 reviewed 137 articles and abstracts (mainly observational studies) on various aspects of female condoms. Based on five randomized trials on effectiveness, the authors concluded that there is limited but convincing evidence that the female condom is effective in increasing protected sex (five studies) and decreasing STI incidence (two studies) among women [21]. Our findings are consistent with theirs. However, our systematic review goes a step further by using GRADE to ascertain the certainty of the evidence. In addition to evaluating the effects of widely marketed condoms, we reviewed the functionality of new female condoms (including the Woman’s, Cupid, Pheonurse, Velvet and Reddy,condoms) based on evidence from more recent trials, and found them to be non-inferior when compared to widely marketed ones.

### Differences between protocol and review

There are several differences between the review protocol and the final review [23]. We had planned to include both randomised and non-randomised trials in this review in order to assess the effectiveness, safety and acceptability of the female condom. However, in this review we included only randomised trials used to assess the effectiveness and safety of the female condom. This was due to the large number of studies identified which explored issues around acceptability. This outcome will be addressed in a separate review. Also, we had planned to include trials that compared the female condom to no treatment or other barrier methods for HIV prevention, for example, male condom, microbicides, diaphragm, vaginal sponges and cervical caps. In this review, we also compared the functionality of different types of new female condoms to existing ones using a non-inferiority analysis. Although the non-inferiority analysis and margins were nor prespecified in our protocol, the risk of bias in this non inferiority analysis is low, considering that the non-inferiority margin is already set by the WHO/UNFPA guidelines [18]. Finally, we had planned to assess the possibility of publication bias by constructing a funnel plot but this was not done due to the small number of studies that reported our outcomes of interest.

### Potential biases in the review process

This systematic review was conducted using methods described in the Cochrane Handbook for systematic reviews of interventions [25] and reported in accordance with the Preferred Reporting Items for Systematic Reviews and Meta-Analyses (PRISMA) items [58]. We are not aware of any biases in the review process. We however highlight that a major limitation of included studies is that condom usage is ascertained based on self-reports by study participants. An objective marker of semen exposure would reduce the risk of recall bias [59].

## Conclusion

The use of female and male condoms (when women are given the option to make a choice of one or both at each sexual act) is more effective than use of male condoms only in preventing chlamydia and gonorrhoea and probably more effective in preventing trichomoniasis and other STIs. There may be no difference between male and female condoms in preventing HIV, however, the certainty of the evidence is low and warrants further research. This research is critical considering that the female condom used in the studies that have provided the evidence base for the effectiveness of the female condom in preventing HIV and STI used the FC1 which is no longer in the market. Such studies should be adequately powered and should equally assess acceptability and adverse events. The advent of new female condoms that may be non-inferior to previous and currently marketed ones provide women with more protection options. Although studies suggest that they may be non-inferior to older female condoms, it is equally important for high quality trials to be conducted that assess the effectiveness of these new condoms in the prevention of HIV and STIs.

## Data Availability

The datasets analysed during the current study are publicly available and were obtained by searching PubMed, Cochrane Central Register of Controlled Trials (CENTRAL), EMBASE, WHO International Clinical Trials Registry Platform (ICTRP) and ClinicalTrials.gov.

## Abbreviations

CENTRAL: Cochrane Central Register of Controlled Trials
FC: Female condom
GRADE: Grading of Recommendations, Assessment, Development and Evaluations
HIV: Human immunodeficiency virus
ICTRP: WHO International Clinical Trials Registry Platform
PATH: Program for Appropriate Technology in Health
STI: Sexually transmitted infections
UNFPA: United Nations Population Fund
USFDA: United States Food and Drug Administration
WHO: World Health Organization
